# Constructing stability: optimal learning in noisy ecological niches

**DOI:** 10.1098/rspb.2024.1606

**Published:** 2024-10-30

**Authors:** Edward D. Lee, Jessica C. Flack, David C. Krakauer

**Affiliations:** ^1^ Complexity Science Hub, Josefstædter Strasse 39, Vienna 1080, Austria; ^2^ Santa Fe Institute, 1399 Hyde Park Rd, Santa Fe, NM 87501, USA

**Keywords:** learning, adaptation, stigmergy, niche construction, scaling, plasticity

## Abstract

Organisms can learn in response to environmental inputs as well as actively modify their environments through niche construction on slower evolutionary time scales. How quickly should an organism respond to a changing environment, and when possible, should organisms adjust the time scale of environmental change? We formulate these questions using a model of learning costs that considers optimal time scales of both memory and environment. We derive a general, sublinear scaling law for optimal memory as a function of environmental persistence. This encapsulates a trade-off between remembering and forgetting. We place learning strategies within a niche construction dynamics in a game theoretic setting. Niche construction is found to reduce or stabilize environmental volatility when learned environmental resources can be monopolized. When learned resources are shared, niche destructors evolve to degrade the shared environment. We integrate these results into a metabolic scaling framework in order to derive learning strategies as a function of body size.

## 1. Introduction

What is the optimal time scale of memory—how long should memory of the environment persist when the environment is changing? And when should the organism invest in influencing the rate of environmental change? Research in a wide range of fields suggests that bidirectional organism–environment feedback through niche construction and symbiosis is common and plays a significant role in shaping evolutionary dynamics. Slowly evolving genes co-evolve with quickly evolving culture [1], as illustrated by the evolution of dairy farming facilitating the selection of alleles for adult lactase persistence [2]. Quickly evolving organisms modify their slowly changing niches and alter selection pressures [3–5], illustrated by yeast modifying fruit environments to attract Drosophilid flies that enhance yeast propagation [6]. To gain information about noisy, hidden variables and reduce social uncertainty, error-prone individual pigtailed macaques (*Macaca nemestrina*) collectively compute a social power structure that reduces uncertainty about the cost of social interaction, making accessible new forms of conflict management [7,8]. Bacterial quorum sensing controls group behaviour in dynamically complex, changing environments [9]. These are diverse examples, but in all of them there is a crucial, shared aspect, which is that the individuals adapt to or learn a changing environment, modify it and are subsequently affected by these changes.

How organisms sense and respond to a changing environment is a prerequisite for understanding evolution, but relevant elements of the problem are scattered across the literature. In their classic work, Berg and Purcell [10] derive physical limits to chemotaxis using dimensional analysis. They show that a bacterium integrating noisy measurements of molecule concentration leads to a scaling relationship for how the fractional error in the estimated concentration Δc/c decreases with the measurement time T as $\Delta c/c∼T^{-1/2}$ [10]. This is part of a long line of work, largely in biophysics, calculating the limits to sensory perception with a fluctuating signal, including the role of adaptive rescaling in neurons under variable light intensity [11], as a problem of efficient coding [12], dynamic concentrations [13] and the role of noise [14]. Despite the parallels, such work remains largely separate from the study of organismal response to environmental cues, or phenotypic plasticity [15,16], in the theoretical biology literature. Plasticity spans behavioural [17], neural [18,19], physiological [20] and immunological [21] changes. Here, a major question is the evolutionary trade-offs inherent in being plastic, balancing the benefits of adapting better to a changing environment against the costs resulting from either possessing this ability or from being less optimal than non-plastic competitors.^1^ Yet, the experimental measurement of the costs and benefits of plasticity is difficult to quantify in complex organisms and so the exact nature of costs remains debated [23]. This speaks to how plasticity is complicated by the appearance of additional scales and elements in the problem. Here, we draw on elements of the two adjacent literatures: we use analytical arguments to derive relationships between time scales and show that learning errors scale inversely with the square root of integration time. We extend the picture to a central, yet largely overlooked, aspect of feedback between organism and the environment [24]; in particular, we incorporate niche construction into a model of agents learning about a noisy and changing environment.^2^

Niche construction refers to the modification of the environment, usually to make it more favourable for survival or reproduction. Beyond the usual formulation, some niche construction strategies are destabilizing by altering or eliminating an environment, and others are stabilizing by fixing the environment. Some social strategies are destabilizing, such as guerrilla warfare in which a weaker party neglects battles by allocating zero resources [25]. In contrast, an example of stabilization is stigmergy in which trails or routes are consolidated through repeated use [26]. More complicated examples include the collective computation of slowly changing power structures in macaque groups [27] and of foraging subgroup size distributions by spider monkeys (*Ateles geoffroyi*) [28]. In the latter, social structures are constructed and then computed through communication and decision networks. Motivated by biological and social examples, we develop a synthetic framework that combines information theory, game dynamics and scaling theory. The framework is used to determine how fast optimal learning interacts with the slower adaptive dynamics of niche construction.

We formulate a simple model that accounts for several time scales. We start by treating learning in terms of the rate of discounting of the past, building a conceptual and mathematical bridge to work on memory [11,29–31]. We take into account four factors: bias, the preference in the environment for a particular state; stability, the rate at which an environment fluctuates [32,33]; precision, the capacity agents have to resolve distinct environmental states; and feedback, the rate of agent modification of the environment. In electronic supplementary material, table S1, we provide examples of studies addressing the interaction of bias, stability and precision. Environmental modification can be either passive or active, where active modification can be destabilizing (increasing the rate of environmental change) as well as stabilizing (decreasing the rate of environmental change). Active agents can stabilize the environment by buffering against variation [5] or slowing its rate of change to reduce uncertainty about the future [7,34]. Finally, we take into account how the precision [35] of an agent’s or organism’s estimates of environmental state influences its ability to fit the environment at a given degree of volatility.

Section 3, ‘Result 1’ examines the conditions under which long memory is beneficial. Section 4, ‘Result 2’ describes the scaling relationship for optimal memory duration and environmental change. Section 5, ‘Result 3’ considers the evolution of active modification with game dynamics. Section 6, ‘Result 4’ compares the costs of poor learning with those of metabolism.

## 2. Material and methods

### (a) Basic model structure and assumptions

**Figure 1.**
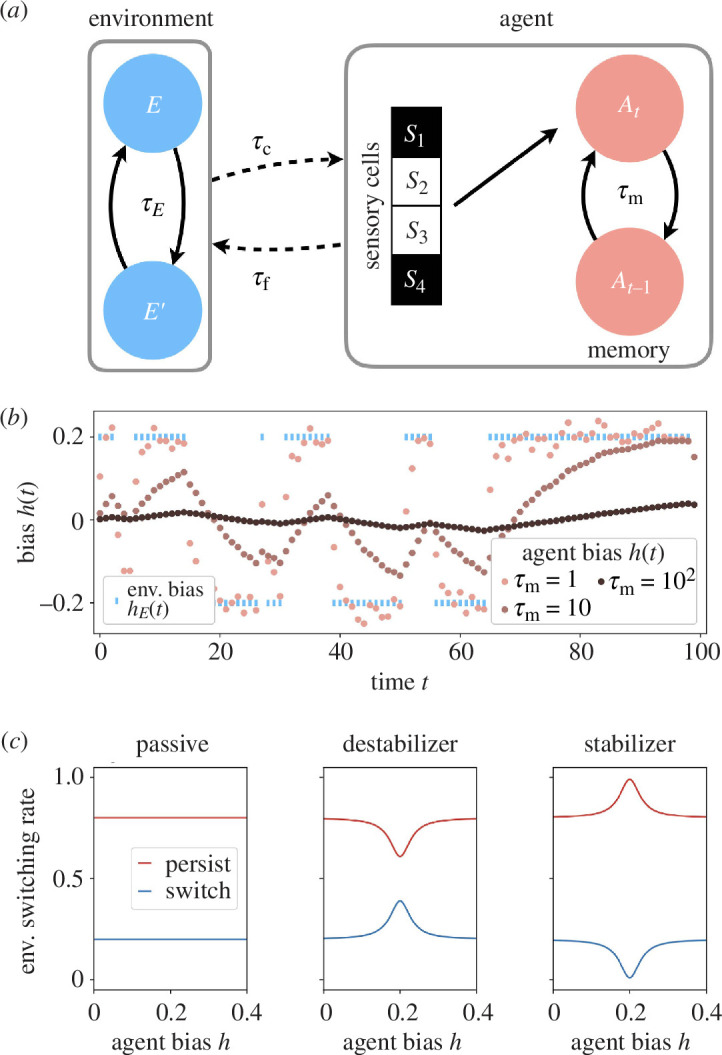
(*a*) Overview of the framework. Environment E switches configuration on time scale $\tau_{E}$ . The agent measures the current environment through sensors with precision $\tau_{c}$ , equivalent to four, black-or-white bits in the example shown. To obtain an estimate of environmental statistics at time t , agent $A_{t}$ combines present sensory estimates with the memory of previous estimates recorded in an aggregator $A_{t-1}$ (equation (2.4)) such that memory decays over time $\tau_{m}$ (equation (2.5)). Coupling with the environment accelerates or decelerates environmental change on time scale $\tau_{f}$ (equation (2.6)). (*b*) Example trajectories of agents learning environmental state $h_{E}(t)$ with short, medium and long memory. (*c*) Rate of environment switching per time step, or the probability, as a function of agent bias h relative to environment bias $h_{E}=0.2$ . For passive agents, the switching rate does not depend on agent bias. For stabilizers α=−0.95 and for destabilizers α=0.95 . For both, v=0.1 from equation (2.6) and the environmental time scale $\tau_{E}=5$ .

We summarize the structure of the model in figure 1, which combines several essential features of a learning agent. The model connects passive agents that learn the statistics of a fluctuating environment with active agents that modify the environment. We summarize the notation in electronic supplementary material, table S2.

The environment E at time t is described by a probability distribution $p_{E}(s,t)$ over configurations s , a vector of descriptive properties. The environment has a bias for preferred states that changes on a relatively slow time scale. Here, we represent the state of the environment with a single bit s∈{−1,1} , analogous to the location of a resource as a choice between left and right [36–40]. In one configuration, the distribution of resources $p_{E}$ is biased to the left at a given time t , or $p_{E}(s=-1,t)>p_{E}(s=1,t)$ , such that an agent with matching preference would do better on average than an agent with misaligned preference. In the mirrored configuration, the environment shows a bias of equal magnitude to the right $p_{E}(s=-1,t)<p_{E}(s=1,t)$ . Such probabilistic bias can be represented as an evolving ‘field’ $h_{E}(t)$ ,


(2.1) $$p_{E}(s,t)=\frac{1}{2}+\frac{s}{2}\tanh h_{E}(t),$$

such that reversal in bias corresponds to the flip of sign $h_{E}(t)\to -h_{E}(t)$ that naturally embodies a symmetry between left and right. At every time point, the environment has a clearly defined bias in one direction or another, determined by setting the external field to either $h_{E}(t)=-h_{0}$ or $h_{E}(t)=h_{0}$ . With probability $1/\tau_{E}$ per unit time, the bias in the environment reverses such that over time $\tau_{E}$ the environment remains correlated with its past. When $\tau_{E}$ is large, we have long correlation times and a slow environment or a ‘slow variable.’ This formulation yields a stochastic environment whose uncertainty depends on both fluctuation rate, such that low rate implies high stability, and the strength of bias for a particular state, such that a strong bias yields a clear environmental signal.

Passive agents sample from the environment and choose a binary action. In principle, the precision of the choice is dependent on the number of sensors contributing to the estimate of environmental state, their sensitivity and the sampling frequency—the contribution of each factor to the estimate can differ. In the model, all the alternatives are captured by $\tau_{c}$ . When $\tau_{c}$ is high (either because the sensors sampled from the environment for a long time, many sensors contributed estimates or each sensor is very sensitive), agents obtain exact measurements of the environment. A small $\tau_{c}$ corresponds to noisy estimates. Thus, $1/\tau_{c}$ is the precision. The resulting estimate of environmental state $\hat{p}$ thus incurs an error $\epsilon_{\tau_{c}}$ ,


(2.2) $$\hat{p}(s,t)=p_{E}(s,t)+\epsilon_{\tau_{c}}(t).$$

From this noisy signal, sensors obtain an estimate of bias $\hat{h}(t)$ , which is the environment bias $h_{E}(t)$ plus measurement noise $\eta_{\tau_{c}}(t)$ ,


(2.3) $$\hat{h}(t)=h_{E}(t)+\eta_{\tau_{c}}(t).$$

In the limit of tight precision, or large $\tau_{c}$ , and given that the noise in the estimated probabilities $\epsilon_{\tau_{c}}(t)$ from equation (2.2) is binomial distributed, the corresponding error in field $\eta_{\tau_{c}}(t)$ converges to a Gaussian distribution (see electronic supplementary material, Text 1). Then, at each time step the agent’s measurement of the environment includes finite-sample noise, which is inversely related to precision.^3^

An aggregation algorithm determines how much to prioritize the current measurement over historical ones. This gives the duration of memory by recording the agent’s estimate of the state of the environment at the current moment in time h(t) and feeding it to sensors at time t+1 with some linear weighting 0≤β≤1 [44],


(2.4) $$h(t+1)=(1-\beta )\hat{h}(t+1)+\beta h(t).$$

This estimate is stored in an ‘aggregator’ $A_{t}$ , and we define h(0)=0 . The weight β determines how quickly the previous state of the system is forgotten, such that when β=0 the agent is constantly learning the new input and has no memory and when β=1 the agent ceases to learn, preserving its initial state. In between, agent memory decays exponentially with lifetime


(2.5) $$\tau_{m}\equiv -1/\log\beta .$$

We think of the weight β that the aggregation algorithm places on the current estimate relative to the stored value as setting agent memory duration $\tau_{m}$ .

The output of this computation is the agent’s behaviour, p(s,t) . We measure the fit to the environment with the divergence between the two probability vectors: one describing the agent and the other the environment. Measures of divergence, like Kullback–Leibler (KL) divergence, and—more generally—mutual information, are considered to be natural measures of goodness of fit in evolutionary and learning dynamics from reinforcement learning through to Bayesian inference [45,46].

Here we extend the model to include feedback by allowing agents to alter environmental stability, which is operationalized as the switching rate.^4^ We add to the unmodified switching rate $1/\tau_{E}$ , the active construction rate,


(2.6) $$\frac{1}{\tau_{f}(t)}\equiv \frac{v^{2}/\tau_{E}}{[h(t)-h_{E}(t){]}^{2}+v^{2}},\;$$

such that the probability q that the environment changes at the next point in time is


(2.7) $$q[h_{E}(t+1)\neq h_{E}(t)]=1/\tau_{E}+\alpha /\tau_{f}(t).$$

Equation (2.6) is written so that it remains normalized for arbitrary v and that the contribution to the total rate gets smaller as the squared distance between agent bias and environment bias $[h(t)-h_{E}(t){]}^{2}$ increases. The probability q of the environment switching to the opposite configuration includes weight α∈(0,1] to tune the strength of destabilizers, or α∈[−1,0) for stabilizers. This means that for positive α , the rate of switching increases as the agent matches the environment more closely to a maximum of double the default environmental switching rate, although particular, finite maximum is not of particular importance. The opposite happens for negative α . The parameter v controls how closely the agent must match the environment to have an effect (i.e. the width of the peak as plotted in figure 1*c*). The two types of active agents capture two ways in which adaptive behaviour can feedforward into the time scale of environmental change.^5^ We note that when α=0 , we obtain passive agents that do not modify their environment, thus connecting passive and active agents to one another along a continuum scale.

Putting these elements together, as shown in figure 1*a*, we obtain a toy agent that learns—but also changes—the statistics of a time-varying and stochastic environment.

## 3. Result 1

### (a) Long memory and rapid learning are favoured when sensors are imprecise and environments are slow

The time scale of memory represents a balance between the costs of preserving an internal state for too long or losing it too quickly. We explore this trade-off by calculating an agent’s fit to a changing environment. The fit can be quantified with the KL divergence between environment $p_{E}(s,t)$ with bias $h_{E}(t)$ and agent p(s,t) ,


(3.1) $$D_{KL}[p_{E}∥p](t)=\sum_{s\in \{-1,1\}}p_{E}(s,t)\log_{2}\left(\frac{p_{E}(s,t)}{p(s,t)}\right).$$

When the KL divergence is $D_{\mathrm{KL}}=0$ , the agents use optimal bet-hedging, known as ‘proportional betting,’ which is relevant to population growth dynamics [47,48]. From another perspective, equation (3.1) is also minimized for Bayesian learners under optimal encoding [49]. Assuming agents are playing a set of games in which they must guess the state of the environment at each time step, equation (3.1) is the information penalty paid by imperfect compared to perfect agents (extensions beyond the logarithmic cost discussed in electronic supplementary material, Text 1).

After averaging over many environment bias switches, we obtain the agent’s typical divergence,


(3.2) $$\bar{D}\equiv \lim_{T\to \infty }\frac{1}{T}\sum_{t=0}^{T-1}D_{KL}[p_{E}∥p](t).$$

The bar notation signals an average over time. Thus, fit improves as $\bar{D}$ decreases.

**Figure 2.**
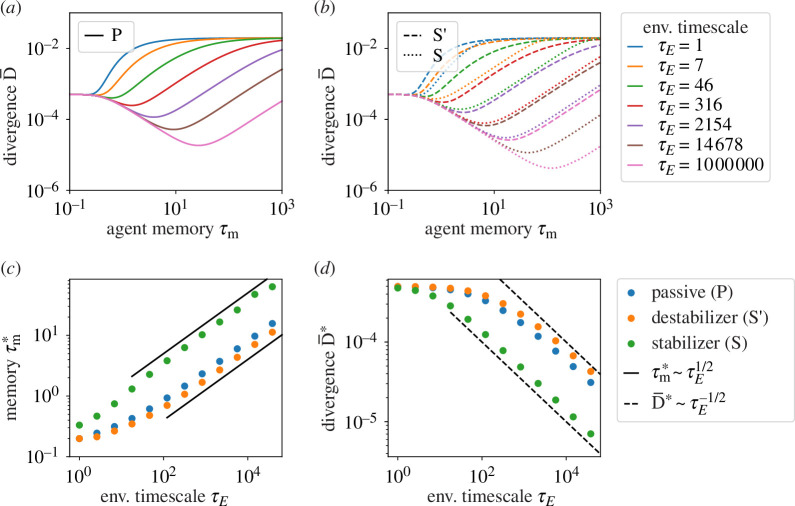
Divergence $\bar{D}$ as a function of agent memory $\tau_{m}$ and environmental time scale $\tau_{E}$ for (*a*) passive and (*b*) active agents including destabilizers $\;S^{\prime }$ and stabilizers  S . For longer $\tau_{E}$ , optimal agents with longer memory always do better as the minima monotonically go down, a pattern emphasized for stabilizers and diminished for destabilizers. (*c*) Scaling of optimal memory duration $\tau_{m}^{*}$ with environmental time scale $\tau_{E}$ , corresponding to minima from (*a*) and (*b*). (*d*) Divergence at optimal memory duration ${\bar{D}}^{*}\equiv \bar{D}(\tau_{m}^{*})$ . Environmental bias $h_{0}=0.2$ ; sensory precision $\tau_{c}^{-1}=10^{-3}$ .

In figure 2*a,b*, we show divergence $\bar{D}(\tau_{m},\tau_{E})$ as a function of the agent’s memory $\tau_{m}$ given environmental time scale $\tau_{E}$ . In the limiting cases in which an agent has either no memory or has infinite memory, the time scale on which environment bias switches ultimately has no effect—we observe convergence across all degrees of bias and stability. When an agent has no memory, or $\tau_{m}=0$ , an agent’s ability to match the environment is solely determined by its sensors. Low precision, or small $\tau_{c}$ , leads to large errors on measured environment bias $h_{E}(t)$ and large divergence $\bar{D}(\tau_{m}=0)$ . On the other hand, high precision increases performance and would depress the intercept $\bar{D}(\tau_{m}=0)$ (electronic supplementary material, equation S5). Moving to the right-hand side of figure 2*a*, for large $\tau_{m}\gg 1$ , behaviour does not budge from its initial state. Assuming that we start with an unbiased agent such that the transition probability is centred as q(h)=δ(h) , the Dirac delta function, the agent’s field is forever fixed at h=0 as we move to the left of figure 2*a,b*. Then, divergence $\bar{D}(\tau_{m}=\infty )$ reduces to a fixed value that only depends on environment bias (electronic supplementary material, equation S6). In between the two limits of zero and infinite agent memory, the model produces a minimum divergence $\bar{D}(\tau_{m}=\tau_{m}^{*})$ as tracked in figure 2*d* as a function of environmental time scale. This indicates the optimal duration of memory $\tau_{m}^{*}$ as in figure 2*c* for a given degree of environment bias and stability.

**Figure 3.**
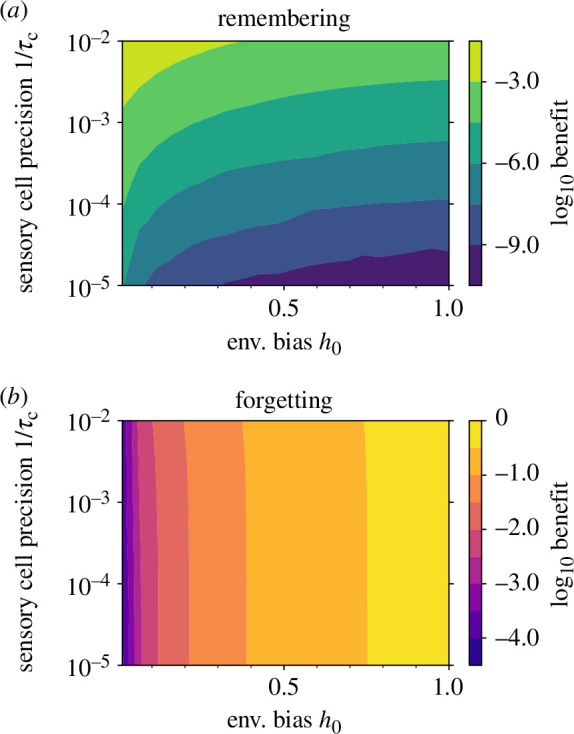
Benefit from (*a*) remembering and from (*b*) forgetting defined as the reduction in divergence at optimal memory duration relative to no memory, $\bar{D}(\tau_{m}=0)-\bar{D}(\tau_{m}^{*})$ , and optimal memory duration to infinite memory, $\bar{D}(\tau_{m}=\infty )-\bar{D}(\tau_{m}^{*})$ , respectively. We show passive agents given environmental time scale $\tau_{E}=10$ . All contours must converge (*a*) when $h_{0}=0$ and (*b*) when $\tau_{c}=0$ . Agent-based simulation parameters are specified in the accompanying code (see electronic supplementary material).

The benefits of memory are more substantial for agents with imprecise sensors. This benefit is the difference $\bar{D}(\tau_{m}=0)-\bar{D}(\tau_{m}=\tau_{m}^{*})$ as shown in figure 3*a*. As one might expect, integrating over longer periods of time provides more of a benefit when the present estimate $\hat{p}$ is noisy, ${\tau_{c}}^{-1}$ is large and sensors are not particularly precise, a deficiency in precision that memory counters by allowing organisms to accumulate information over time. This intuition, however, only applies in the limit of large environment bias $h_{0}$ where the contours of optimal memory flatten and become orthogonal to precision ${\tau_{c}}^{-1}$ . When the bias in the environment is weak, the curved contours show that the benefits of memory come to depend strongly on the nontrivial interaction of precision and environment bias. The complementary plot is the benefit from forgetting, $\bar{D}(\tau_{m}=\infty )-\bar{D}(\tau_{m}=\tau_{m}^{*})$ in figure 3*b*, which is largely determined by bias $h_{0}$ . When the bias is strong, the costs of estimating the environment inaccurately are large, and it becomes important to forget, if sensors are imprecise. Thus, the model encapsulates the trade-off between remembering and forgetting and indicates the emergence of simple cost contours in the limits of high environment bias and high sensory precision.

## 4. Result 2

### (a) Memory scales sublinearly with environmental persistence time

For sufficiently slow environments, or sufficiently large $\tau_{E}$ , we find that optimal memory duration $\tau_{m}^{*}$ scales with the environmental time scale $\tau_{E}$ sublinearly as in figure 2*c*. To derive the scaling between optimal memory and environmental time scale, we consider the limit when agent memory duration is small relative to the environment $\tau_{m}\ll \tau_{E}$ . Under this condition, optimal memory represents a trade-off between a poor fit lasting time $\tau_{m}$ as the agent learns and then a good fit for time $\tau_{E}-\tau_{m}$ . During the learning phase, the agent pays a typical cost at every single time step and the cost is of order $\tau_{m}$ such that the cost rate grows linearly with its duration, $C\tau_{m}$ , for constant *C *. When the environment is stable, agent precision is enhanced by a factor of $\tau_{m}$ because the agent effectively averages over $\tau_{c}\tau_{m}$ random samples, or a gain of $G\;\log\;\tau_{m}+H$ for constants G and H .^6^ When we weight each term by the fraction of time spent in either transient or stable phases, $\tau_{m}/\tau_{E}$ and $(\tau_{E}-\tau_{m})/\tau_{E}$ , respectively, we obtain the trade-off


(4.1) $$C\frac{\tau_{m}^{2}}{\tau_{E}}-\frac{\tau_{E}-\tau_{m}}{\tau_{E}}\left(G\log\tau_{m}+H\right).$$

Each of the respective terms is measured in units of bit rate. At optimal memory $\tau_{m}^{*}$ , equation (4.1) will have zero derivative with respect to $\tau_{m}$ . Keeping only the dominant terms and balancing the resulting equation, we find


(4.2) $$\tau_{m}^{*}∼\tau_{E}^{1/2}$$

(see electronic supplementary material, Text 1 for details). The scaling argument aligns with numerical calculation as shown in figure 2*c*; it is accurate for relatively large ratios of $\tau_{m}/\tau_{E}$ and generalizes to multidimensional and asymmetric environments (see electronic supplementary material, Text 2 for details and generalization beyond logarithmic costs).

Similarly, we calculate how optimal divergence ${\bar{D}}^{*}$ scales with environmental time scale. Assuming that the agent has a good estimate of the environment such that the error in average configuration $\epsilon_{\tau_{c}}(t)$ is small, agent behaviour is $p_{E}(s,t)+\epsilon_{\tau_{c}}(t)$ and $\epsilon_{\tau_{c}}(t)$ is normally distributed. Then, we expand the divergence about $p_{E}(s,t)$ in Taylor series of error $\epsilon_{\tau_{c}}(t)$ (electronic supplementary material, text 1). Given the measurements that would be taken over a time scale of $\tau_{m}^{*}$ , the precision of this estimate is further narrowed by a factor of $\tau_{m}^{*}$ such that


(4.3) $${\bar{D}}^{*}∼1/\tau_{m}^{*}∼\tau_{E}^{-1/2}.$$

Although we do not account for the transient phase, we expect the relation in equation (4.3) to dominate in the limit of large $\tau_{E}$ , and the numerical calculations indeed approach the predicted scaling in figure 2*d*. In contrast, when the environment does not fluctuate, or bias $h_{0}=0$ , agents pay no cost for failing to adapt to new environments and infinite memory is optimal (assuming that agents start without bias). Overall, the sublinear scaling between memory duration and the rate of environmental change indicates an economy of scale. Optimal agents require proportionally less memory in slow environments than would be true under a linear relationship.

## 5. Result 3

### (a) A ratchet for slower time scales

We explore the conditions under which active modification of the environment can evolve. To do so, we consider the stability of pure populations to invasion by other types of agents as a function of variation in agent memory. According to the formulation, a pure population is susceptible to invasion if the divergence of the new population is lower than that of the incumbent population. Among passive agents, there is one unique optimal agent memory $\tau_{m}^{*}$ having fixed other agent properties: a pure population is stable against alternative passive strategies. The case is not so simple when active agents modify the environment by changing the environmental time scale, thereby displacing the dominant population from the optimal point.

For example, a pure population of stabilizing agents S with optimal time scale $\tau_{m}^{*}$ for an environmental time scale is unstable to the spontaneous mutation of a few to passive agents. This is because when a small fraction ϵ of S is replaced with passive agents P, the introduction reduces the stabilization effect such that the new environmental switching rate is increased by the amount $\epsilon \alpha /\tau_{f}(t)$ . This implies that the new optimal memory decreases by an amount proportional to that quantity when ϵ≪1 . Assuming that the invading P conform to the new optimal time scale, they will have lower divergence than the original S population. This is always the case because the divergence is convex about the optimal time scale, so any population at the optimum will always displace any that is not. In this case, the new optimum is yet again unstable because P grows, ϵ increases and $\tau_{E}$ shifts lower, permitting invasion by yet another population of passive agents $P^{\prime }$ that have a smaller memory $\tau_{m}$ . This continues until all stabilizers have been outcompeted, whereupon we return to the original observation of a pure passive population. Thus, while it is the case that stabilizers are better adapted to the environment, sharing it with passive agents leads to an evolutionary dead end in a way that mimics the usual ‘tragedy of the commons’ argument [50] as it is displaced.^7^

**Figure 4.**
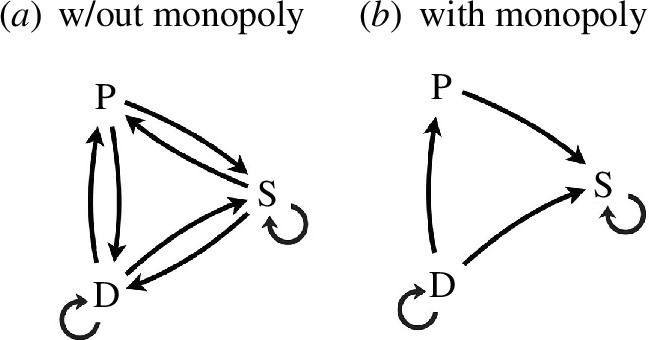
Susceptibility of pure populations of passive agents (P), destabilizers (D) and stabilizers (S) to invasion. Outgoing arrows indicate to which competitor the originating population is unstable.

On the other hand, a pure population of passive agents can be invaded by stabilizers. This is possible because the appearance of ϵ fraction of S in the total population will slow down the environmental time scales by an amount $\epsilon \alpha /\tau_{f}(t)$ , in which the S do best assuming that they are sitting at the optimal memory. Since the increase in ϵ leads to a slower time scale, this benefits stabilizers with a longer agent memory. Such ratcheting continues until we have a pure S population at the optimal agent memory that is required by their stabilization effect. Such a population is, however, susceptible to invasion by P according to the previous argument, so there is no general stable outcome but only ones that depend on precise agent parameters. When we consider all the pairwise invasions of pure populations as we show in figure 4*a*, we find that nearly all are possible (including S invaded by another S and D invaded by another D). This is a result of the general fact that the divergence is convex about optimal memory and almost all pure populations can be invaded by any other type; there is no generally stable outcome in the overall dynamics that we have just considered.

Environmental stabilization, however, is a stable strategy if individuals can monopolize the benefits of a slowed time scale. This is to say that stabilizers do not share the same time scale as their competitors but experience the slowed time scales from monopolized niche construction alone. In the natural world, the monopolization of resources from the stabilized environment could occur through physical encryption (e.g. undetectable pheromones [51]), the erasure of signal (e.g. food caching [52]) or restricting social information (e.g. concealment [53]).

In this case, the evolutionary dynamics is much simpler as we show in figure 4*b*, and a pure population of stabilizers emerges. This is because, in any given environment, stabilizers can always lower the divergence by slowing the local time scale. Furthermore, stabilizers ratchet their niche construction to increasingly higher levels because slower stabilizers always lower the divergence. In contrast, D is unstable relative to P, S and weaker D agents when returning our attention to figure 4*a*, making them an evolutionary dead end. This is not true if D commits all competitors to the same destabilized environment—a kind of forced commiseration—because a global reduction in environmental time scales displaces other populations from the optimal memory point, which is occupied by the invading D (not shown). In short, destabilizers must be able to degrade the shared environment to successfully invade, whereas stabilizers must be able to hoard the benefits of niche construction.^8^

## 6. Result 4

### (a) Learning costs dominate short lifetimes, metabolic costs dominate long lifetimes

We compare the scaling relation for learning costs with metabolic costs. To make the connection, we start with a simple assumption that the lifetime of an organism T can be broken up into a number of environmental episodes that have duration $\tau_{E}$ , or that


(6.1) $$T\propto \tau_{E}.$$

Equation (6.1) is the statement that an organism experiences in its lifetime a typical number of switches in the environment, and so its lifetime is proportional to the duration of each episode.

Then, we take into account the well-documented observation that physical constraints on circulatory networks responsible for energy distribution influence organismal traits across the animal kingdom from mice to blue whales, including lifespan and size [54–56]. These relationships correspond to predictions from metabolic scaling theory such as the relation between metabolic rate B and mass, $B\propto M^{3/4}$ [57], also known as Kleiber’s law.

**Figure 5.**
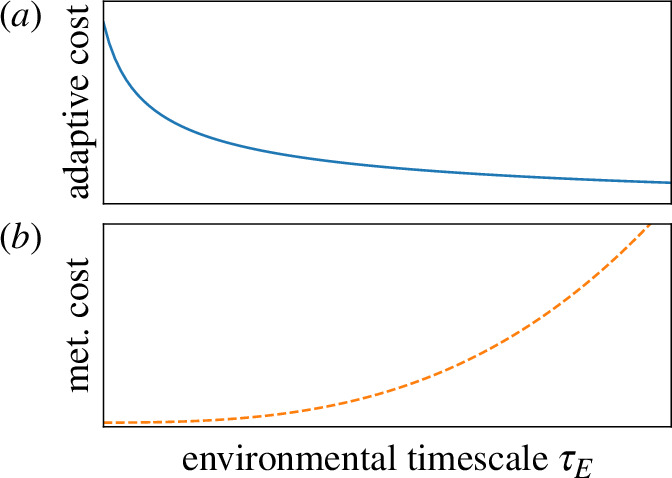
Scaling of adaptive and metabolic costs with environmental time scale $\tau_{E}$ . (*a*) Adaptive cost $\bar{D}$ is largest at small $\tau_{E}$ , but (*b*) metabolic costs are largest for longer-lived organisms.

From metabolic scaling theory, we know that if lifetime grows sublinearly with mass as $T\propto M^{1/4}$ , then $B\propto T^{3}\propto \tau_{E}^{3}$ . From equation (4.3), we obtain the relationship $B\propto ({\bar{D}}^{*}{)}^{-6}$ . This means that the learning cost decreases as the metabolic cost increases, as we show in figure 5.^9^ The competing scalings of adaptive and metabolic costs suggest that for small organisms (or those in transient environments) the former will make a disproportionate contribution to their lifetime energy budget. This is consistent with observations on developmental neural growth in butterflies, where the adaptive gain is measurable in the constitutive investment in brain mass [19] in contrast with the importance of metabolic scaling for larger mammals.^10^

## 7. Discussion

Life is adaptive and optimal phenotypic plasticity through learning would seem to depend on a diversity of properties of both organism and environment (electronic supplementary material, table S1). We show that this need not be the case by organizing different aspects of the adaptive process into a unified framework for time scales and a largely overlooked aspect of niche construction (figure 1). With a combination of analytic and numerical techniques, we derive and validate a general scaling law that optimal time scale of memory duration scales sublinearly with environmental rates of change, with or without niche construction (figure 2). The scaling originates from the fact that the persistence of memory penalizes agents that cannot adapt quickly to new environments. This result can be generalized to environments with asymmetric statistics (beyond the symmetric case considered here and detailed in electronic supplementary material, Text 2) and to more than two environments. The result does not rely specifically on the linear learning rule in equation (2.4) but only depends on the existence of a memory time scale and a proportional cost. As a corollary, the information error (or the KL-divergence, ${\bar{D}}^{*}$ ) decreases with agent memory $\tau_{m}^{*}$ at optimality, ${\bar{D}}^{*}∼\tau_{m}^{*}$ . These generalizations support the conjecture that the scaling law may be observed across many examples of niche construction.

Using the model as the basis for evolutionary dynamics, we consider the stability of populations that are optimally adapted to a given environmental time scale. In this case, the stabilizers reduce environmental volatility. Although this improves fit to the environment, it is an evolutionarily unstable strategy. This is because competitors that share the same environment all benefit from stabilization and slowly come to displace the active stabilizers under steady-state conditions. One possible consequence of such an iterated dynamics is the extinction of stabilizing niche construction. We find, however, that stabilizing niche construction can evolve as agents push the environment to ever slower time scales if they are able to monopolize benefits: the stable environment needs to be owned. By contrast, agents that make the environment more volatile can dominate only if they can force competitors to pursue costly learning.

Notably, slow environmental time scales seem to characterize many of the properties that we associate with niche construction—whether ant pheromone trails, food caching, collectively computing power structures, writing or map-making. These constitute forms of niche construction that promote the stability or predictability of the local environment [58] and reduce the number of environmental configurations that an organism needs to encode in a suitable policy. Detailed experiments indicate that organisms are sensitive to environmental correlations and display corresponding phenotypic adaptations [20,59], but how organisms might leverage niche construction to change such time scales remains an intriguing experimental question.

In real systems, a problem with the rise of stabilizing niche construction is that it creates a public good that reduces environmental uncertainty and provides a benefit to all agents. This can be exploited by free riders—it produces a tragedy of the commons [50]. By contrast, destabilization emerges as a natural mathematical complement to stabilization in the framework. An interesting variation on the idea is the recent Reddit–Gamestop event in which powerful hedge funds are thought to have introduced volatility to markets by manipulating Reddit users to short-squeeze yet other hedge funds [60]—although the hedge funds earned a return, in contrast with the collective loss implied by the model. Destabilizing strategies deserve deeper study as complementary strategies to the canonical stability perspective on niche construction.

There is a clear connection between environmental stability and cognitive repertoire. How might the emergence of slow time scales promote the development of simple or complex cognitive strategies? We note that the increase in environmental time scales (lower variability) reduces the required temporal complexity of the agent, in agreement with other simulations [24]. This might allow agents to devote more of the lifetime energy budget to other kinds of cognitive complexity. This hypothesis is consistent with related work on institutions and social structure as a form of collectively encoded memory [27,61–63] or as devised constraints [64] that slow down the need to acquire functional information. In a pigtailed macaque society [7], individuals collectively compute a social-power distribution from status signalling interactions. The distribution of power as a coarse-grained representation of underlying fight dynamics changes relatively slowly and consequently provides a predictable social background against which individuals can adapt. By reducing uncertainty and cognitive costs, the power distribution facilitates the emergence of novel forms of impartial conflict management. Conflict management, in turn, further reduces volatility, allowing individuals to build more diverse and cohesive local social niches and engage in a greater variety of socially positive interactions [65]. In other words, outsourcing memory to a stable social structure encoded in the power distribution allows for a significant increase in social complexity. More generally, we anticipate that one of the features of slowing environmental time scales might be to free proportionally more cognitive resources and facilitate the emergence of new functions [66,67].

Beyond cognitive complexity, our work relates directly to a rich and evolving literature on phenotypic plasticity [24,68]. While the trade-off between the gain from having plasticity versus the many potential associated costs of having such an ability is widely modelled, explicit connections between the models in their trade-offs are missing. We provide a concise mathematical formulation of such a trade-off in equation 4.1 that makes a testable prediction in terms of quantities, or time scales, that are applicable to both simulation and experiment. Thus, the model provides a way to consider together the results in the literature because we derive aspects that may be independent of the details of the particular system in the scaling regime (equation (4.2)). Importantly, the argument can be easily extended to consider different kinds of costs beyond the assumptions we make here. As we emphasize, a key and elucidating aspect is the exponents in determining how the costs and benefits trade off with one another. Often, the focus is on the values of the constants in front of the terms that are being balanced in experiments [23]. This suggests a shift in perspective to complement ongoing attempts to connect the interdisciplinary literature [69].

As an example of one such extension, we place adaptive, potentially cognitive, strategies within the larger energy budget of a life history strategy. For example, memory requires investing in neural tissue, which incurs metabolic and constitutive costs [18]. Under the simplest of assumptions about how organism lifetime relates to environmental time scale, we find that learning costs peak for short-lived organisms, whereas metabolic costs grow with lifespan. We provide a first-order and general prediction for how the terms should scale and a baseline for considering experiments on agents navigating noisy environments. One particularly relevant example is the ability of marine life to adapt to changing environmental variation: direct comparison with experiments presents potential fruitful directions to map the features of our model—such as environmental time scale (or autocorrelation in salinity [20] and acidity [70]) and agent memory (or behavioural flexibility [71] and phenotypic differentiation [72]). Thus, the framework provides a way of connecting niche construction with agent behaviour, generates testable predictions and highlights constraints relating to adaptive strategies that complement the metabolic laws of life.

## Data Availability

Supplementary material is available online [73].

## Footnotes

^1^
Extensions beyond costs and limits include game-theoretic dynamics on the adoption of plastic or non-plastic strategies [22].

^2^
In evolutionary biology, ‘learning’ and ‘plasticity’ are considered to be processes that occur within organism lifetimes, whereas ‘adaptation’ is an evolutionary process. This norm is not always the case in biophysics. We will generally try to stick to the evolutionary biology language but will use forms of ‘adapt’ more loosely.

^3^
We do not consider intrinsic or induced costs [41] for measurement of the environment or in §2a for memory. The associated machinery required for learning is costly [42] and so is the computation itself [43]. Some of these are incorporated implicitly into our metabolic costs later.

^4^
In principle, niche construction could also affect the bias of the environment as well as the switching rate. For the case of foraging in a large, homogeneous environment, however, an individual agent would alone have little impact on the distribution of resources as captured by the bias. This motivates our focus on the switching rate to capture the effects of niche construction.

^5^
Note that in this binary example, the new environmental configuration when switching is unique, enforcing a deterministic switch, but in general, there may be a large number of *K* >> 1 options such that the agent cannot easily guess the results of environmental fluctuations.

^6^
Note that the logarithmic gain comes from considering the KL divergence between the environment and the estimate by the agent in equation (3.1) at each moment in time *t*. The equation reduces to a term proportional to the error in the bias $\eta_{\tau_{c}}(t)$ and the dominant factor is the coefficient in front of the exponential in electronic supplementary material, Eq S2.

^7^
Another possible co-evolutionary equilibrium is that S reduces its memory time scale to compensate for the reduced environmental time scale that comes from the shared population of P. In this case, however, the S is again open to invasion by P with a slower time scale, leading to S’s eventual extinction.

^8^
Here, we focus on optimality in terms of agent memory but not precision. In general, increased agent precision permits agents to do better for a fixed agent memory. However, better precision usually comes with an increased investment in sensory tissue and computation, which can negate its benefits.

^9^
The inverse relationship holds even if we generalize equation (6.1) to allow for a changing environmental time scale with lifetime, or $T∼\tau_{E}^{\phi }$ for positive exponent ϕ and ϕ=1 is the case we have just considered. This would capture, for example, a change in the number of environmental cycles with organism lifetime such as, when ϕ<1/2 , a longer memory time scale relative to lifetime and consistent with the aphorism ‘an elephant never forgets.’ This leads to the generalized scaling $B∼({\bar{D}}^{*}{)}^{-6\phi }$ . However, this implies that learning costs are outweighed by metabolic ones for longer- lived organisms. If learning costs do matter, it must be the case that either ϕ is very small or negative, which we generally do not expect.

^10^
As noted in the cited study and its citations, experience leads to a larger brain size, indicating that learning from such experience is sufficiently valuable to warrant concomitant constitutive and induced costs.
